# The Effects of Attention Pre-Allocation and Target-Background Integration on Object-Based Attention

**DOI:** 10.1371/journal.pone.0119414

**Published:** 2015-03-19

**Authors:** Fengpei Hu, Changyong Jiao, Songpo Zhao, Huahua Dong, Xiao Liu, Yuji Yi, Jun Wang

**Affiliations:** 1 College of Economics and Management, Zhejiang University of Technology, Hangzhou, Zhejiang, People’s Republic of China; 2 Department of Psychology, Zhejiang Sci-Tech University, Hangzhou, Zhejiang, 310018, China; 3 New York State Psychiatric Institute, New York, NY, 10032, United States of America; 4 Department of Psychiatry, UT Southwestern Medical Center, Dallas, TX, 75390, United States of America; University Medical Center Goettingen, GERMANY

## Abstract

Object-based attention has been documented as an important mechanism with which to control attention in several studies. To date, two main hypotheses have been proposed to interpret object-based attention: attention spreading and prioritization of search. There is evidence that supports these hypotheses in the literature. In the present study, we sought to compare these two hypotheses systematically by manipulating two factors: the integration of the target and background and the presence of attention pre-allocation. For this purpose, we used a flanker task in which the location of the task-relevant target was fixed, but the relationship between the target and the background varied. In addition, attention pre-allocation was presented in only half of the conditions. The results revealed that the attention spreading hypothesis was supported only when attention was not pre-allocated and target-background integration was high; however, the prioritization hypothesis was supported in all other conditions. Our findings provide insight into the comparisons of the attention spreading and prioritization hypotheses. Furthermore, our findings suggest that attention resources may be the underlying factor determining appropriate strategy in the control of attention.

## Introduction

Individuals are exposed to enormous amounts of information daily. Attention allows individuals to select task-relevant information and ignore irrelevant information, particularly during complex visual cognition tasks. Control of attention can be either space- [1] or object-based [2,3]. Space-based attention suggests that attention is allocated to a specific region, while object-based attention suggests that attention is allocated to objects. When compared to space-based attention, object-based attention is suited to more complex visual scenes (e.g., overlapping images [4]) but is relatively smaller (restricted to the object boundaries) and less robust [5].

Egly et al. [2] proposed one of the most frequently used paradigms with which to explore object-based attention. In their study, two rectangles were presented apart from the central fixation, either vertically or horizontally. Following a short delay, a cue appeared at one end of one of the rectangles to direct the observer’s attention to that location. The simple onset target followed the cue, and observers were asked to produce a rapid response to the onset of the target. The critical manipulation used in this study was the target location. Most of the targets (75% of trials) occurred in the cued location (valid cue); however, they could also occur at the opposite end of the cued rectangle (invalid cue, same-object condition) or in the other rectangle at the same distance from the cue (invalid cue, different-object condition). Participants detected the invalid same-object target more quickly than they detected the invalid different-object target, a finding that indicates an object-based component of attention.

Object-based attention has also been widely supported in other literature including studies involving both humans [6–9] and animals [10–13]. Moreover, recent neuroimaging studies have suggested that object-based attention may occur at the early perceptual stage of processing [14–15]. For example, activities in early visual areas (e.g., V1) were found to be modulated by changes in the attended object rather than unattended objects or distractors [12,16].

Object-based attention researchers have proposed several hypotheses regarding the way in which processing differs for attended objects and unattended objects [17]. The attentional spreading view of object-based attention proposes that an object-based attention effect arises from the spread of attention within an object’s boundaries [18]. This involuntary spreading process facilitates and enhances sensory processing of all of the parts and features of a single object [19]. Contrary to the attentional spreading hypothesis, the attentional shifting hypothesis [20] suggests that the spread of attentional resources across the whole object (i.e., the sensory enhancement theory) is not necessarily a mandatory process and occurs only when attentional shifting is required. For example, Lamy and Egeth [20] failed to report an object-based attention effect when targets were presented simultaneously. Another possible outcome of object-based attention is prioritization [21]. According to the prioritization hypothesis, object-based attention affects the target’s priority in the sequence of the search. As a result, an attended object, including both the attended and unattended portions, will be searched prior to an unattended object. In the current study, we focused primarily on the comparison between prioritization and attentional spreading.

Both the prioritization and attention spreading hypotheses have received experimental support [2,7,22]. However, less is known about which hypothesis best accounts for the object-based attention effect. Shomstein and Yantis [23] addressed this issue using a “flanker” task paradigm. In their study, three rectangles were presented to participants for 1000 ms, followed by three letters. The three letters were presented in either the central rectangle (same-object condition) or all three rectangles (different-object condition). Participants were instructed to respond to the center letter (target) while ignoring the peripheral letters (flankers). The key manipulation was that the participants always knew the location of the target, and no visual search was needed. Previous studies have shown that responses to central targets were affected by task-irrelevant flankers connected to the targets [24]. Flankers that were associated with the same response as the target (compatible condition) facilitated the response, whereas flankers associated with a different response to that of the target (incompatible condition) affected the response. Therefore, if attention spreads within an attended object and stops at the borders (thereby enhancing the sensory perception of that object), one would expect a larger flanker compatibility effect in the same-object condition relative to that of the different-object condition. In contrast, the prioritization hypothesis would predict a comparable flanker compatibility effect in both conditions, as the target would only be presented in the center, and no display search would be needed. The results of the study supported the prioritization hypothesis. Importantly, this study was limited, as the target stimuli (letters) may have been too salient and may not have been perceived as an intrinsic part of the rectangle; this may have resulted in participants failing to display perceptual-level attention spreading when distinguishing the central letter from the peripheral letters. Richard and colleagues [25] conducted a similar flanker paradigm to address this limitation by using concavities, instead of letters, as the targets and the flankers. Participants were required to respond to the shape of the central concavity (rectangular or circular). The results from this study indicated that there was a significantly larger flanker compatibility effect in the same-object condition relative to the different-object condition, thereby providing strong support for the attention spreading hypothesis.

Two possible critical factors could underlie the discrepancies between these two studies: the integration of the target-background object and the effect of attention pre-allocation. Specifically, Shomstein and Yantis [23] used low target-background integration stimuli (letters), while high target-background integration stimuli (concavities) were used in the study conducted by Richard et al. [25]. Indeed, it has recently been shown that this may affect the attention spreading process [26]. Therefore, it would be optimal to investigate the effect of target-background integration using identical stimuli. In addition, background rectangles were presented 1000 ms prior to the target letters in Shomstein and Yantis’s study. During this time, attention may already have spread through the entire rectangle; therefore, participants could narrow their attention to the target location for the upcoming target recognition task [25]. Indeed, in experiment 4 of the study conducted by Richard et al., the object-based attention effects were absent when the presenting background rectangle was added for 1000 ms, similar to the procedures in Shomstein and Yantis’s study [23].

**Fig 1:**
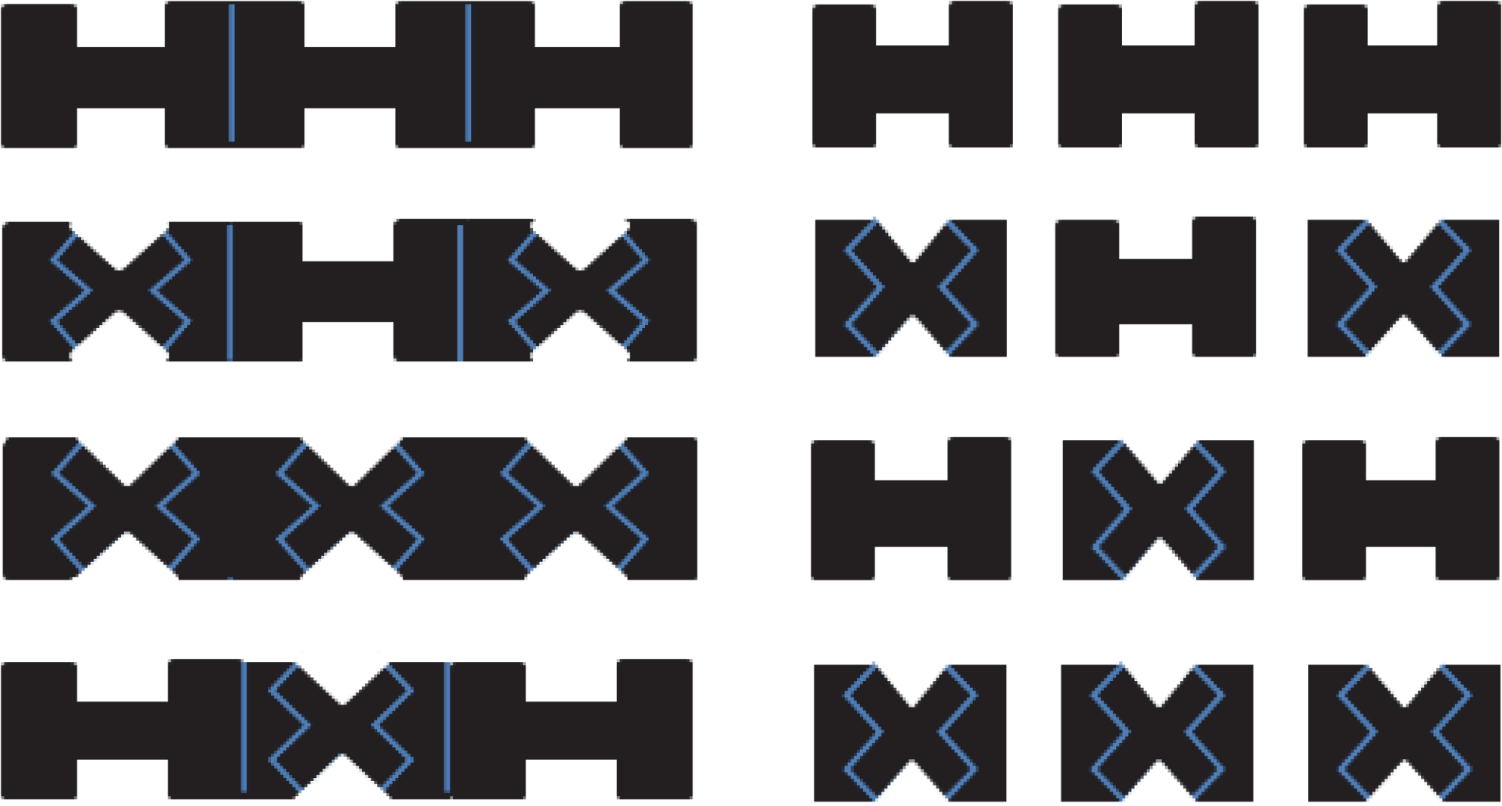
Illustration of the stimuli. The participants were required to judge either the letter or shape of the center bite. Left: target and flankers are in the same object. Right: target and flankers are in different objects.

**Fig 2:**
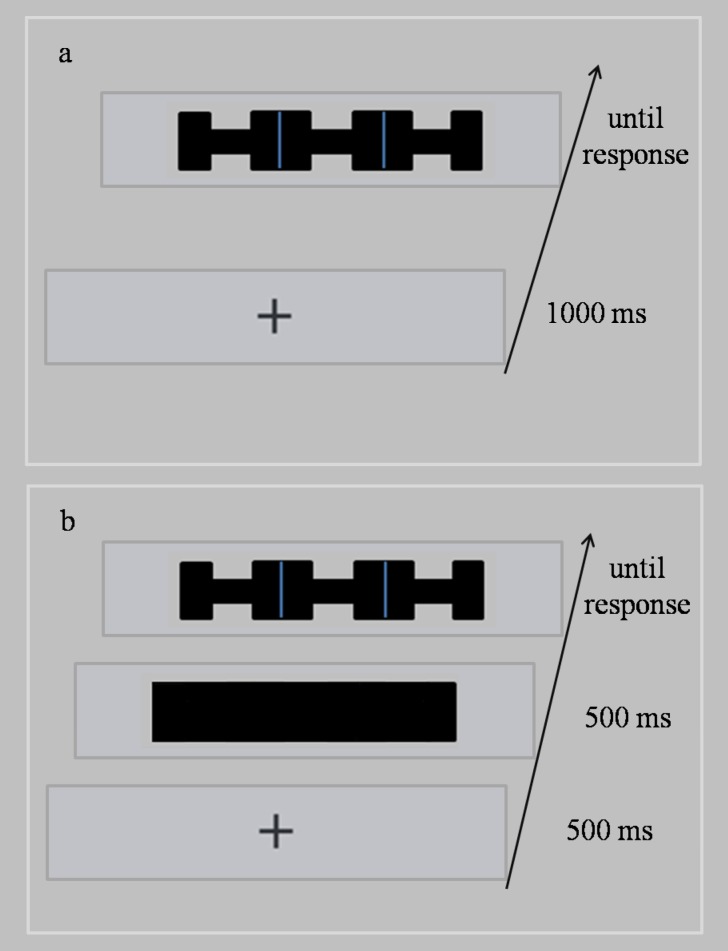
Schematic diagram of the tasks completed. a: Experiments 1A and 1B. b: Experiments 2A and 2B (same-object condition). The gray rectangles used in this Fig. were only for visual display purposes; they were not actually presented on the screen. Stimuli within one rectangle were presented on one screen. In addition, Fig. 2B is an illustration of the same-object condition, whereas the three small rectangles were presented for 500 ms in the different-object condition.

Several studies, including the research conducted by Richard et al., have compared the prioritization and attention spreading hypotheses by investigating the effects of target-background integration and attention pre-allocation[27,28]; however, a systemic investigation using identical stimuli is necessary to address the inconsistent findings in the existing literature. In the present study, a similar flanker paradigm to those used in the studies conducted by Shomstein and Yantis [23] and Richard et al. [25] was implemented; however, we combined the low (letter) and high (concavity) integration targets within the same object (see Fig. 1). Participants were instructed to respond to the letter in Experiment 1A and the shape of the concavity in Experiment 1B (Fig. 2A). Similar tasks were conducted in Experiments 2A and 2B; however, the presentation of a background rectangle was presented for 500 ms to serve as attention pre-allocation (Fig. 2B). Based on the literature, we expected a larger flanker compatibility effect in the same-object condition, relative to the different-object condition, during high target-background integration (Experiment 1B). We expected the same flanker compatibility effects with low target-background integration (Experiment 1A). However, we expected the interaction between compatibility and object type to be absent when participants were given sufficient attention pre-allocation time, regardless of the level (high or low) of target-background integration (Experiments 2 A& 2B).

## Experiments 1A and 1B

### Methods

#### Participants

Seventy undergraduates from Zhejiang University of Technology with normal or corrected-to-normal vision participated in the experiments (Experiment 1A: 35 participants, 19 men; Experiment 1B: 35 participants, 14 men). Participants were compensated for their participation. The ethics committee of Zhejiang University of Technology approved the study. All participants provided their written informed consent prior to participation. One participant in Experiment 1B failed to complete the experiment; therefore, his data were excluded from the analysis.

#### Stimuli

The task stimuli are shown in Fig. 1. Participants were seated 60 cm away from a monitor. The stimuli contained either one large rectangle (same-object condition) or three small rectangles (different-objects condition). The large rectangle extended 1.5° × 10° of the visual angle, and the small rectangles extended 1.5° × 3° of the visual angle. The three small rectangles were aligned horizontally and separated by gaps measuring 1.5° × 0.5°. The target and flanker pairs were either the letter “X” with triangular bites or the letter “H” with rectangular bites. The bites were 1° wide × 0.5° deep. The target was always presented at the center, and the far edges of the flankers were 5° away from the center.

#### Procedures

Each trial began with a 1000 ms fixation cross. Thereafter, the stimulus, with target and flanker pairs, was presented until the participant responded (Fig. 2A). In experiment 1A, participants were asked to press a key (“X” or “H”) to report the central letter. Half of the trials included an “X” and the remaining trials included an “H.” In experiment 1B, participants were asked to determine whether the shape of the center bite was rectangular or triangular, by pressing the corresponding keys (“1” for rectangular, “2” for triangular). Half of the targets were rectangular and the other half were triangular. In both experiments, targets were consistent with flankers in half of the trials and inconsistent in the other half of the trials. Feedback was provided to the participants during the practice sessions but was not provided during the experimental sessions. There were 12 blocks with 12 trials each. Each participant completed eight unanalyzed practice trials at the beginning of the experiment.

## Results and Discussion

The overall accuracy for all of the conditions in Experiments 1A and 1B was high (see Table 1 for details), indicating that participants accomplished the task successfully. The RTs for the different conditions were of primary interest. The RTs that were over three standard deviations above or below the mean were removed from the analysis; this resulted in a total of 1.8% of the trials being excluded. A two-way repeated measures ANOVA was used to examine the effects of object type (same-object, different-object) × compatibility (compatible, incompatible) on RT data.

**Table 1:** Mean accuracy (in percentages) for each experiment.

Experiment	Same object		Different object	
	Compatible	Incompatible	Compatible	Incompatible
1A	98.3	97.6	97.5	97.2
1B	97.4	98.0	97.6	97.8
2A	98.4	96.9	98.4	96.9
2B	97.5	95.1	96.4	94.8

### Experiment 1A

**Fig 3:**
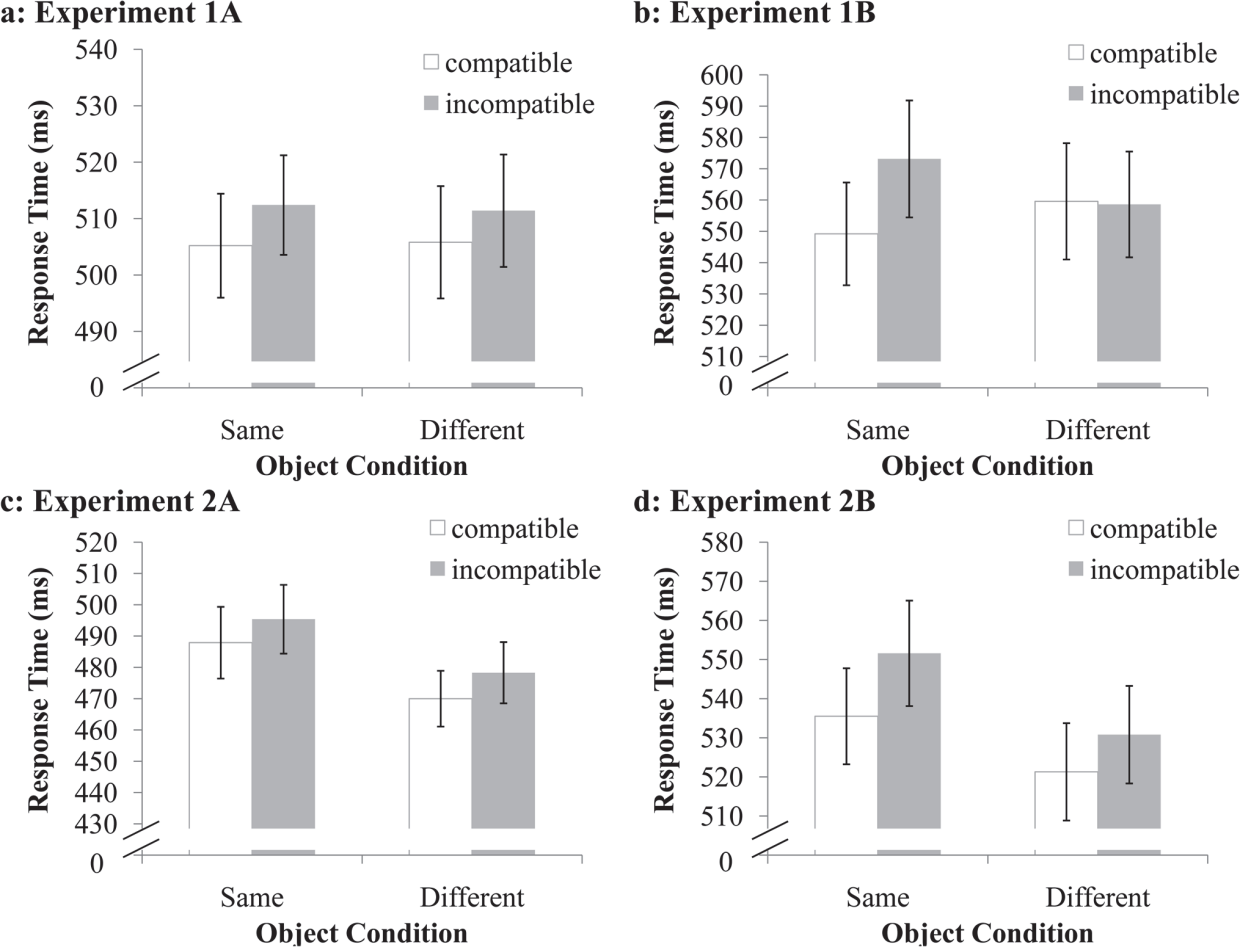
Mean reaction times (plus standard error). Note that the response time ranges differed between these four subfigures.

The object type × compatibility ANOVA revealed a significant main effect for compatibility, *F*(1, 34) = 7.95, MSE = 181.94, *p* < .01, with faster responses in the compatible condition (505 ms) relative to the incompatible condition (511 ms; Fig. 3A). The main effect of object type was nonsignificant, *F*(1, 34) = 0.00, MSE = 368.65, *p* = .951. The interaction between compatibility and object type was also nonsignificant, *F*(1, 34) = 0.09, MSE = 240.24, *p* = .762; this was the most important analysis of Experiment 1A.

### Experiment 1B

The object type × compatibility ANOVA revealed a main effect for compatibility, *F*(1, 33) = 12.32, MSE = 362.25, *p* < .001, with longer RTs in the incompatible condition (565 ms) relative to the compatible condition (554 ms). The main effect of object type was nonsignificant, *F*(1, 33) = 0.17, MSE = 889.70, *p* = .684. Contrary to other experiments, the interaction between compatibility and object type was significant, *F*(1, 33) = 7.01, MSE = 755.64, *p* < .05. Post hoc analyses indicated that the compatibility effect was significant in the same-object condition, *p* < .001 but nonsignificant in the different-object condition, *p* = .860 (Fig. 3B).

To strengthen the findings of Experiment 1, we also analyzed the results of Experiments 1A and 1B in an overall ANOVA, with the integration factor added as a between-subjects variable. The results indicated a three-way interaction for object type, compatibility, and integration, *F*(1, 67) = 4.77, MSE = 494.10, *p* < .05. This finding suggests that different integration targets influenced the attention process.

The results reported in Experiment 1 indicated that when participants were asked to pay attention to a low integration target (“X” or” H” in Experiment 1A), a prioritization effect was exhibited, as there was no interaction between object type and compatibility. This is consistent with the results of experiment 5 in the study conducted by Richard et al. However, with the use of the same stimuli and participants being asked to attend to the high integration target (concaves, Experiment 1B), the pattern of results supported an attentional spreading account of object-based attention. This was demonstrated by the exhibition of the flanker compatibility effect only in the same-object condition. Therefore, the integration between an attended target and an object background played an important role in the effects of object-based attention.

## Experiments 2A and 2B

### Methods

#### Participants

Sixty-one undergraduates from Zhejiang University of Technology with normal or corrected vision participated in the study; participants received compensation for their participation (Experiment 2A: 31 participants, 16 men; Experiment 2B: 30 participants, 14 men). The ethics committee of Zhejiang University of Technology approved the study. All participants provided their written informed consent prior to participation.

#### Stimuli and procedures

The displays used in Experiments 2A and 2B were the same as those used in Experiments 1A and 1B. In addition, the procedure was identical to that used in Experiments 1A & 1B, with the following exceptions (see Fig. 2B). The background objects were presented for 500 ms following the presentation of the fixation cross for 500 ms. The background objects were either a large black rectangle (same-object condition) or three small black rectangles (different-object condition); these were the same size as the stimuli. Three letters with concaves then appeared on the background objects until the participants responded. Participants were asked to respond to letters in Experiment 2A and concaves in Experiment 2B.

### Results and Discussion

The overall accuracy for all of the conditions in Experiments 2A and 2B was high (also see Table 1 for details). The behavioral results for Experiments 2A and 2B are shown in Fig. 3. Similar to the previous experiments, RTs that were three standard deviations above or below the mean were removed from the analysis; this trimming resulted in 0.9% of the trials being excluded. Thereafter, a two-way repeated measures ANOVA was performed to examine the effects of object type (same-object, different-object) × compatibility (compatible, incompatible) on RT data.

#### Experiment 2A

The object type × compatibility ANOVA revealed a significant main effect for compatibility, *F*(1, 30) = 4.88, MSE = 398.89, *p* < .05 (see Fig. 3C); the RTs in the compatible condition (478 ms) were shorter relative to the incompatible condition (486 ms). The results also revealed a significant main effect of object type, *F*(1, 30) = 12.94, MSE = 732.29, *p* < .001; the RTs were shorter when the flankers were displayed in different objects (474 ms) relative to when they were displayed in the same object (491 ms). However, the interaction between compatibility and object type was nonsignificant, *F*(1, 30) = 0.03, MSE = 227.59, *p* = .869.

#### Experiment 2B

In Experiment 2B, an object type × compatibility ANOVA was performed. A main effect of compatibility was observed (Fig. 3D), *F*(1, 29) = 6.89, MSE = 718.78, *p* < .05, with faster responses in the compatible condition (528 ms) relative to the incompatible condition (541 ms). The main effect for object type was also significant, *F*(1, 29) = 22.68, MSE = 405.94, *p* < .01; responses were faster in the different-object condition (526 ms) relative to the same-object condition (544 ms). The interaction between compatibility and object type was nonsignificant, *F*(1, 29) = 0.50, MSE = 663.60, *p* = .486.

As in Experiment 1, we analyzed the results of Experiments 2A and 2B in an overall ANOVA, with the integration factor added as a between-subjects variable. The results indicated that there was no significant three-way interaction for object type, compatibility, and integration, *F*(1,59) = 0.49, MSE = 441.90, *p* = .487; this was consistent with our prediction.

We also combined Experiments 1 and 2 to further confirm the role of pre-allocation in object-based attention. In particular, we added pre-allocation as the between-subjects variable and analyzed the results of Experiments 1A and 2A and Experiments 1B and 2B. The results indicated that there was no significant three-way interaction between object type, compatibility, and pre-allocation when Experiments 1A and 2A, in which the low integration stimuli (letter) were used, were combined, *F*(1,64) = 0.11, MSE = 234.31, *p* = .741; this was consistent with our hypothesis. Moreover, it is important to note that the lack of a three-way interaction for Experiments 1B and 2B, *F*(1,62) = 1.88, MSE = 712.59, *p* = .175, was unexpected. The lack of an interaction effect may have occurred due to the confounding effect of the between-subjects design. To test this possibility, we conducted an additional experiment by asking 22 participants to perform both Experiments 1B and 2B. The results indicated a significant main effect of object type, *F*(1,21) = 20.58, MSE = 4588.83, *p* < .001, with faster responses in the different-object condition (506 ms) relative to the same-object condition (516 ms). There was also a main effect of compatibility, *F*(1,21) = 5.03, MSE = 408.83, *p* < .05, with faster responses in the compatible condition (508 ms) relative to the incompatible condition (515 ms). The main effect of pre-allocation was nonsignificant, *F*(1,21) = 0.57, MSE = 16168.03, *p* = .460, and the three-way interaction between object type, compatibility, and pre-allocation was marginally significant, *F*(1,21) = 3.01, MSE = 570.64, *p* < .1.

Based on the pattern of findings, we ruled out the attention spreading hypothesis, as no significant interactions between object types and compatibility were observed in Experiments 2A and 2B. Rather, these results supported the prioritization hypothesis. In addition, in Experiments 2A and 2B, responses were slower when the target and the flankers appeared within the same object relative to when they appeared in different objects. A similar magnitude of object-type effect (same vs. different) has been observed in previous studies [23,25]; however, the effect was relatively small (18 ms). This small object-type effect may reflect a segmentation process in which participants need to separate the flankers from the target in the same-object condition; however, such separation is not required during the different-object condition. Importantly, this finding must be interpreted with caution, as similar object-type effects were not replicated in Experiments 1A and 1B. One possible explanation for this contradictory finding is that attention was only pre-allocated to the object’s background in Experiments 2A and 2B. In the different-object condition, attention was focused on the small center rectangle; however, in the same-object condition, attention was deployed to the large rectangle. Given limited attentional resources, attention allocated to the central location is denser in the different-object condition relative to the same-object condition. As a result, slower responses were observed in the same-object condition.

## General Discussion

The objective of this study was to compare the attention spreading and prioritization hypotheses systematically while manipulating two key factors: integration between the target and the object background and the pre-allocation of attention. Four experiments provided evidence in favor of a prioritization effect when there was low integration between the target and the object background, or attention was pre-allocated to the object background; this conclusion was drawn, as there were no interactions between compatibility and object type (Experiments 1A, 2A, and 2B). The attention spreading effect was present only when there was high integration between the target and the object background and no attention pre-allocation to the object background (Experiment 1B). Importantly, identical stimuli were used for the comparison; therefore, this ruled out possible confounding factors due to differences in the perceptual features of the stimuli.

### The role of target-object integration

In Experiments 1A and 2A, an attention spreading effect was always absent when there was low integration between the target and the object background; this was independent of whether there was a time lapse between the background object and the target. This pattern of findings is consistent with the results reported by Richard et al. (Experiments 4 and 5) [25]. As addressed by Richard et al., the letters used as stimuli may not have been perceived as integral parts of the background object; therefore, they may not have contributed to the shapes of the objects. Therefore, a complete object representation would not have been constructed. Previous studies have suggested that the quality of object representations is critical for object-based attention [29]; it is also important in the spatial spread of top-down facilitation within object boundaries. In addition, the strength of the object-based attention effect could be modulated based on how strongly features are integrated into an object. For example, in Marino and Scholl’s study [30], stronger object-based attention effects were observed in objects with closed contours relative to objects with open contours.

### The role of pre-allocation attention

Based on the aforementioned literature, one reasonable prediction is that stimuli with high target-background integration generate a larger flanker compatibility effect in same-object conditions relative to different-object conditions; this supports the attention spreading hypothesis. However, only the results from Experiment 1B confirmed this prediction. When there was a time lapse (500 ms) between the background object and target (Experiment 2B), the object-based attention effect disappeared; this supports the prioritization hypothesis. When the findings from Experiments 1B and 2B were combined with those from previous studies (e.g., see [25], in which low target-background integration stimuli [letters] were used), it appeared that there may have been a serial process in which attention operated subsequent to the completion of object representation. When sufficient time was provided for attention pre-allocation to the object and completion of its representation, the process of attention worked in a prioritizing way. This occurred regardless of whether there was high or low target-background integration. In other words, the attention prioritization process may occur at a late stage; this notion has been discussed in previous studies (for review see [31]). In contrast, when the target and object background were presented simultaneously (Experiments 1A and 1B), object representation and attention operated in a parallel fashion, which was similar to the findings of other studies (e.g., object-based attention was influenced by an object configuration change even after attention had been cued to it.) [32,33]. Due to limited resources, prioritizing may be involved during low (Experiment 1A), but not high (Experiment 1B) target-background integration conditions.

### Strategic control

Similar to the findings of a previous study [34], neither attention prioritization nor attention spreading could account for all of the findings of the four experiments. Therefore, object-based attention may be the “default” model for attention when available attentional resources are limited [35]. However, when there are sufficient attentional resources, participants may choose the best strategy (e.g., prioritization) to perform the task [32,36]. When a highly integrated object and target appeared at the same time (Experiment 1B), participants were forced to attend to the object and target simultaneously. Therefore, they did not have sufficient attentional resources to initiate a strategy other than the automatic spreading of attention along the object boundary. When there was sufficient time allowed to perceive the object first (Experiments 2A, 2B) or there was low target-object integration (Experiments 1A, 2A), the participants may have had sufficient attentional resources to recruit a different strategy, such as prioritization. In addition, factors other than attentional resources may also influence strategy choice, including past experiences [5,6], expectations [8,23,37,38], and task demands [20,32,35,39].

In conclusion, in the present study, we demonstrated that target-object integration and attention pre-allocation are two key factors in object-based attention. Moreover, a resource-dependent strategic control mechanism may underlie human choice of the optimal attention control strategy.
